# Supplementary material for: Development of a New Indirect ELISA Test for the Detection of Anti-Feline Coronavirus Antibodies in Cats
Source: Vet Sci. 2025 Mar 4;12(3):245. doi: 10.3390/vetsci12030245 (PMC11946180; doi:10.3390/vetsci12030245)
Supplement: Supplementary file 1 [file vetsci-12-00245-s001.zip › vetsci-3454671-supplementary.pdf]

Supplementary Table S1. Preliminary ELISA results on Nunc MaxiSorp plate.
Absorbance values obtained on Nunc MaxiSorp microplates coated with 0, 2.5, 5, 10, and 20 µg/mL of FCoV antigen diluted in CB buffer pH 9.6 or in PBS 1X and blocked in PBS containing 1% BSA. The conjugate antibody was tested at dilutions ranging from 1:5,000 to 1:20,000. Samples: one positive, one negative (dilution 1:200, 50 µL/well). n.d.: not determined.

Sample	FCoV antigen (µg/mL)	Coating: CB buffer pH 9.6				Coating: PBS			
		1:5,000	1:10,000	1:20,000	0	1:5,000	1:10,000	1:20,000	0
Positive	20.0	1.149	n.d.	n.d.	n.d.	n.d.	n.d.	n.d.	n.d.
	10.0	0.902	0.397	0.224	0.044	0.718	0.361	0.221	0.043
	5.0	0.718	0.274	0.161	0.041	0.456	0.251	0.161	0.041
	2.5	0.271	0.163	0.100	0.042	0.257	0.152	0.098	0.040
	0	0.097	0.068	0.054	0.041	0.103	0.079	0.056	0.040
Negative	20.0	0.104	n.d.	n.d.	n.d.	n.d.	n.d.	n.d.	n.d.
	10.0	0.104	n.d.	n.d.	n.d.	n.d.	n.d.	n.d.	n.d.
	5.0	0.107	n.d.	n.d.	n.d.	n.d.	n.d.	n.d.	n.d.
	0	0.099	n.d.	n.d.	n.d.	n.d.	n.d.	n.d.	n.d.

Supplementary Table S2. Performance of FCoVCHECK Ab ELISA at sample dilutions 1:100 or 1:200 against IFAT as reference assay. Number of samples analysed: 110. Discordant results are highlighted in bold and marked with an asterisk. OD: optical density. POS: positive; NEG: negative.

N. sample	IFAT	Sample dilution 1:200		Sample dilution 1:100	
		OD	Results	OD	Results
1	NEG	0.167	NEG	0.105	NEG
2	NEG	0.117	NEG	0.129	NEG
3	NEG	0.083	NEG	0.079	NEG
4	NEG	0.102	NEG	0.097	NEG
5	NEG	0.073	NEG	0.082	NEG
6	NEG	0.070	NEG	0.070	NEG
7	NEG	0.062	NEG	0.061	NEG
8	NEG	0.055	NEG	0.052	NEG
9	NEG	0.221	NEG	0.237	NEG
10	NEG	0.058	NEG	0.053	NEG
11	NEG	0.095	NEG	0.096	NEG
12	NEG	0.068	NEG	0.066	NEG
13	NEG	0.076	NEG	0.063	NEG
14	NEG	0.058	NEG	0.062	NEG
15	NEG	0.074	NEG	0.067	NEG
16	NEG	0.108	NEG	0.123	NEG
17	NEG	0.054	NEG	0.051	NEG
18	NEG	0.074	NEG	0.081	NEG
19	NEG	0.059	NEG	0.063	NEG
20	NEG	0.077	NEG	0.083	NEG
21	NEG	0.095	NEG	0.090	NEG
22	NEG	0.162	NEG	0.180	NEG
23	NEG	0.073	NEG	0.067	NEG
**24***	**POS**	**0.097**	**NEG**	**0.110**	**NEG**
25	NEG	0.059	NEG	0.062	NEG
26	NEG	0.246	NEG	0.241	NEG
27	NEG	0.071	NEG	0.070	NEG
28	NEG	0.077	NEG	0.075	NEG
**29***	**POS**	**0.078**	**NEG**	**0.070**	**NEG**
30	NEG	0.088	NEG	0.072	NEG
31	NEG	0.096	NEG	0.105	NEG
32	NEG	0.143	NEG	0.157	NEG
33	POS	0.589	POS	0.694	POS
34	POS	1.332	POS	1.434	POS
35	NEG	0.220	NEG	0.262	NEG
36	NEG	0.109	NEG	0.117	NEG
37	NEG	0.114	NEG	0.141	NEG
38	POS	0.771	POS	0.865	POS
39	POS	0.282	POS	0.371	POS
40	NEG	0.090	NEG	0.098	NEG
41	POS	0.493	POS	0.551	POS
42	NEG	0.079	NEG	0.086	NEG
**43***	**POS**	**0.192**	**NEG**	0.308	POS
44	POS	0.727	POS	0.735	POS
45	NEG	0.076	NEG	0.068	NEG
46	POS	0.409	POS	0.494	POS
47	POS	0.362	POS	0.591	POS
48	POS	0.418	POS	0.385	POS
49	POS	0.562	POS	0.662	POS

Continued on the next page

Supplementary Table S2 *(continued)*

N. sample	IFAT	Sample dilution 1:200		Sample dilution 1:100	
		OD	Results	OD	Results
50	POS	0.418	POS	0.447	POS
51	NEG	0.153	NEG	0.189	NEG
52	POS	0.465	POS	0.567	POS
53	NEG	0.233	NEG	0.289	NEG
54	POS	1.452	POS	1.455	POS
55	NEG	0.099	NEG	0.088	NEG
**56***	**POS**	**0.178**	**NEG**	0.316	POS
57	POS	1.651	POS	1.599	POS
58	NEG	0.188	NEG	0.253	NEG
59	POS	1.065	POS	1.116	POS
60	NEG	0.163	NEG	0.120	NEG
61	POS	0.617	POS	0.660	POS
**62***	**POS**	**0.171**	**NEG**	0.309	POS
63	NEG	0.117	NEG	0.145	NEG
64	NEG	0.168	NEG	0.215	NEG
65	POS	0.740	POS	0.768	POS
66	POS	0.385	POS	0.416	POS
**67***	**POS**	**0.082**	**NEG**	**0.085**	**NEG**
68	POS	1.790	POS	1.507	POS
69	NEG	0.064	NEG	0.066	NEG
70	POS	0.699	POS	0.876	POS
**71***	**POS**	**0.194**	**NEG**	0.311	POS
**72***	**POS**	**0.220**	**NEG**	0.349	POS
**73***	**POS**	**0.128**	**NEG**	0.363	POS
74	NEG	0.146	NEG	0.205	NEG
**75***	**POS**	**0.190**	**NEG**	0.318	POS
**76***	**POS**	**0.078**	**NEG**	**0.088**	**NEG**
77	POS	1.337	POS	1.421	POS
**78***	**POS**	**0.237**	**NEG**	0.305	POS
79	POS	1.003	POS	1.124	POS
80	POS	1.162	POS	1.095	POS
81	POS	0.709	POS	1.011	POS
82	POS	0.376	POS	0.388	POS
83	POS	1.061	POS	1.195	POS
84	NEG	0.092	NEG	0.108	NEG
85	POS	0.269	POS	0.435	POS
86	POS	0.369	POS	0.549	POS
87	POS	0.528	POS	0.625	POS
88	POS	0.465	POS	0.519	POS
89	POS	0.542	POS	0.650	POS
**90***	**POS**	**0.213**	**NEG**	0.313	POS
91	POS	1.500	POS	1.376	POS
92	POS	0.939	POS	0.883	POS
93	NEG	0.072	NEG	0.083	NEG
94	NEG	0.075	NEG	0.089	NEG
95	POS	0.268	POS	0.337	POS
**96***	**POS**	**0.132**	**NEG**	0.335	POS
97	POS	0.801	POS	0.808	POS
98	POS	0.292	POS	0.323	POS
99	POS	0.811	POS	0.995	POS

Continued on the next page

Supplementary Table S2 *(continued)*

N. sample	IFAT	Sample dilution 1:200		Sample dilution 1:100	
		OD	Results	OD	Results
100	POS	1.216	POS	1.032	POS
101	POS	0.723	POS	0.727	POS
102	POS	1.781	POS	1.497	POS
103	POS	1.123	POS	1.064	POS
104	POS	0.506	POS	0.575	POS
105	POS	1.230	POS	1.236	POS
106	POS	1.183	POS	1.112	POS
107	POS	1.298	POS	1.074	POS
**108***	**POS**	**0.205**	**NEG**	0.304	POS
109	POS	1.605	POS	1.431	POS
110	POS	2.095	POS	1.733	POS

Supplementary Table S3. Positive and negative controls selection. Results are expressed as mean OD ± SD. PC: positive control formed by a mixture of anti-FIPV antibody and protein G-HRP. NC: negative control, sample diluent. Negative samples: 48 samples tested at dilution 1:100. Good results, selected for costs and performances reasons, are highlighted in bold and marked with an asterisk; n: number of tests.

Samples	N.	Anti-FIPV antibody	Protein G-HRP	Results (OD ± SD)
PC	**1***	1:300	1:500	3.338 ± 0.045 (n=4)
	2	1:600	1:500	2.561 ± 0.043 (n=2)
	**3***	1:600	1:800	2.631 ± 0.016 (n=2)
	4	1:600	1:1,200	2.426 ± 0.005 (n=2)
	5	1:600	1:1,800	2.238 ± 0.038 (n=2)
	**6***	1:800	1:300	2.632 ± 0.066 (n=2)
	**7***	1:800	1:500	2.630 ± 0.016 (n=2)
	8	1:800	1:800	2.472 ± 0.039 (n=2)
	9	1:800	1:1,000	2.152 ± 0.015 (n=2)
	10	1:1000	1:500	2.098 ± 0.002 (n=2)
	11	1:1000	1:1,000	2.022 ± 0.021 (n=2)
	12	1:1000	1:2,000	1.051 ± 0.004 (n=2)
NC	-	-	-	0.043 ± 0.002 (n=24)
Negative samples	-	-	-	0.114 ± 0.063 (n=48)

Supplementary Table S4. Cut-off determination. Sp: Specificity; Se: Sensitivity; PPV: positive predictive value; NPV: negative predictive value; LR+: positive likelihood ratio; LR-: negative likelihood ratio; J: Jouden's index.

N. Cut-off	OD cut-off	Sp	1 - Sp	Se	Se-Sp	J	VPP	VPN	Accuracy	LR +	LR -	J max
1	0.050	0.000	1.000	1.000	1.000	0.000	0.564	-	0.564	1.000	-	0.935
2	0.060	0.063	0.938	1.000	0.938	0.063	0.579	1.000	0.591	1.067	0.000	
3	0.070	0.292	0.708	0.984	0.692	0.276	0.642	0.933	0.682	1.389	0.055	
4	0.080	0.375	0.625	0.984	0.609	0.359	0.670	0.947	0.718	1.574	0.043	
5	0.090	0.521	0.479	0.952	0.431	0.472	0.720	0.893	0.764	1.986	0.093	
6	0.100	0.604	0.396	0.952	0.347	0.556	0.756	0.906	0.800	2.404	0.080	
7	0.110	0.667	0.333	0.935	0.269	0.602	0.784	0.889	0.818	2.806	0.097	
8	0.120	0.688	0.313	0.935	0.248	0.623	0.795	0.892	0.827	2.994	0.094	
9	0.130	0.750	0.250	0.935	0.185	0.685	0.829	0.900	0.855	3.742	0.086	
10	0.140	0.750	0.250	0.935	0.185	0.685	0.829	0.900	0.855	3.742	0.086	
11	0.150	0.792	0.208	0.935	0.144	0.727	0.853	0.905	0.873	4.490	0.081	
12	0.160	0.813	0.188	0.935	0.123	0.748	0.866	0.907	0.882	4.989	0.079	
13	0.170	0.813	0.188	0.935	0.123	0.748	0.866	0.907	0.882	4.989	0.079	
14	0.180	0.813	0.188	0.935	0.123	0.748	0.866	0.907	0.882	4.989	0.079	
15	0.190	0.854	0.146	0.935	0.081	0.790	0.892	0.911	0.900	6.415	0.076	
16	0.200	0.854	0.146	0.935	0.081	0.790	0.892	0.911	0.900	6.415	0.076	
17	0.210	0.875	0.125	0.935	0.060	0.810	0.906	0.913	0.909	7.484	0.074	
18	0.220	0.896	0.104	0.935	0.040	0.831	0.921	0.915	0.918	8.981	0.072	
19	0.230	0.896	0.104	0.935	0.040	0.831	0.921	0.915	0.918	8.981	0.072	
20	0.240	0.917	0.083	0.935	0.019	0.852	0.935	0.917	0.927	11.226	0.070	
21	0.250	0.938	0.063	0.935	-0.002	0.873	0.951	0.918	0.936	14.968	0.069	
22	0.260	0.958	0.042	0.935	-0.023	0.894	0.967	0.920	0.945	22.452	0.067	
23	0.270	0.979	0.021	0.935	-0.044	0.915	0.983	0.922	0.955	44.903	0.066	
24	0.280	0.979	0.021	0.935	-0.044	0.915	0.983	0.922	0.955	44.903	0.066	
25	0.290	1.000	0.000	0.935	-0.065	0.935	1.000	0.923	0.964	-	0.065	
26	0.300	1.000	0.000	0.935	-0.065	0.935	1.000	0.923	0.964	-	0.065	
27	0.310	1.000	0.000	0.887	-0.113	0.887	1.000	0.873	0.936	-	0.113	
28	0.320	1.000	0.000	0.806	-0.194	0.806	1.000	0.800	0.891	-	0.194	
29	0.330	1.000	0.000	0.790	-0.210	0.790	1.000	0.787	0.882	-	0.210	
30	0.340	1.000	0.000	0.758	-0.242	0.758	1.000	0.762	0.864	-	0.242	
31	0.350	1.000	0.000	0.742	-0.258	0.742	1.000	0.750	0.855	-	0.258	
32	0.360	1.000	0.000	0.742	-0.258	0.742	1.000	0.750	0.855	-	0.258	
33	0.370	1.000	0.000	0.726	-0.274	0.726	1.000	0.738	0.845	-	0.274	
34	0.380	1.000	0.000	0.710	-0.290	0.710	1.000	0.727	0.836	-	0.290	
35	0.390	1.000	0.000	0.677	-0.323	0.677	1.000	0.706	0.818	-	0.323	
36	0.400	1.000	0.000	0.677	-0.323	0.677	1.000	0.706	0.818	-	0.323	
37	0.410	1.000	0.000	0.677	-0.323	0.677	1.000	0.706	0.818	-	0.323	
38	0.420	1.000	0.000	0.661	-0.339	0.661	1.000	0.696	0.809	-	0.339	
39	0.430	1.000	0.000	0.661	-0.339	0.661	1.000	0.696	0.809	-	0.339	
40	0.440	1.000	0.000	0.645	-0.355	0.645	1.000	0.686	0.800	-	0.355	
41	0.450	1.000	0.000	0.629	-0.371	0.629	1.000	0.676	0.791	-	0.371	
42	0.460	1.000	0.000	0.629	-0.371	0.629	1.000	0.676	0.791	-	0.371	
43	0.470	1.000	0.000	0.629	-0.371	0.629	1.000	0.676	0.791	-	0.371	
44	0.480	1.000	0.000	0.629	-0.371	0.629	1.000	0.676	0.791	-	0.371	
45	0.490	1.000	0.000	0.629	-0.371	0.629	1.000	0.676	0.791	-	0.371	
46	0.500	1.000	0.000	0.613	-0.387	0.613	1.000	0.667	0.782	-	0.387	
47	0.510	1.000	0.000	0.613	-0.387	0.613	1.000	0.667	0.782	-	0.387	
48	0.520	1.000	0.000	0.597	-0.403	0.597	1.000	0.658	0.773	-	0.403	
49	0.530	1.000	0.000	0.597	-0.403	0.597	1.000	0.658	0.773	-	0.403	
50	0.540	1.000	0.000	0.597	-0.403	0.597	1.000	0.658	0.773	-	0.403	
51	0.550	1.000	0.000	0.581	-0.419	0.581	1.000	0.649	0.764	-	0.419	
52	0.560	1.000	0.000	0.565	-0.435	0.565	1.000	0.640	0.755	-	0.435	
53	0.570	1.000	0.000	0.548	-0.452	0.548	1.000	0.632	0.745	-	0.452	

Continued on the next page

Supplementary Table S4 *(continued)*

N. Cut-off	OD cut-off	Sp	1 - Sp	Se	Se-Sp	J	VPP	VPN	Accuracy	LR +	LR -
54	0.580	1.000	0.000	0.532	-0.468	0.532	1.000	0.623	0.736	-	0.468
55	0.590	1.000	0.000	0.532	-0.468	0.532	1.000	0.623	0.736	-	0.468
56	0.600	1.000	0.000	0.516	-0.484	0.516	1.000	0.615	0.727	-	0.484
57	0.610	1.000	0.000	0.516	-0.484	0.516	1.000	0.615	0.727	-	0.484
58	0.620	1.000	0.000	0.516	-0.484	0.516	1.000	0.615	0.727	-	0.484
59	0.630	1.000	0.000	0.500	-0.500	0.500	1.000	0.608	0.718	-	0.500
60	0.640	1.000	0.000	0.500	-0.500	0.500	1.000	0.608	0.718	-	0.500
61	0.650	1.000	0.000	0.500	-0.500	0.500	1.000	0.608	0.718	-	0.500
62	0.660	1.000	0.000	0.468	-0.532	0.468	1.000	0.593	0.700	-	0.532
63	0.670	1.000	0.000	0.452	-0.548	0.452	1.000	0.585	0.691	-	0.548
64	0.680	1.000	0.000	0.452	-0.548	0.452	1.000	0.585	0.691	-	0.548
65	0.690	1.000	0.000	0.452	-0.548	0.452	1.000	0.585	0.691	-	0.548
66	0.700	1.000	0.000	0.435	-0.565	0.435	1.000	0.578	0.682	-	0.565
67	0.710	1.000	0.000	0.435	-0.565	0.435	1.000	0.578	0.682	-	0.565
68	0.720	1.000	0.000	0.435	-0.565	0.435	1.000	0.578	0.682	-	0.565
69	0.730	1.000	0.000	0.419	-0.581	0.419	1.000	0.571	0.673	-	0.581
70	0.740	1.000	0.000	0.403	-0.597	0.403	1.000	0.565	0.664	-	0.597
71	0.750	1.000	0.000	0.403	-0.597	0.403	1.000	0.565	0.664	-	0.597
72	0.760	1.000	0.000	0.403	-0.597	0.403	1.000	0.565	0.664	-	0.597
73	0.770	1.000	0.000	0.387	-0.613	0.387	1.000	0.558	0.655	-	0.613
74	0.780	1.000	0.000	0.387	-0.613	0.387	1.000	0.558	0.655	-	0.613
75	0.790	1.000	0.000	0.387	-0.613	0.387	1.000	0.558	0.655	-	0.613
76	0.800	1.000	0.000	0.387	-0.613	0.387	1.000	0.558	0.655	-	0.613
77	0.810	1.000	0.000	0.371	-0.629	0.371	1.000	0.552	0.645	-	0.629
78	0.820	1.000	0.000	0.371	-0.629	0.371	1.000	0.552	0.645	-	0.629
79	0.830	1.000	0.000	0.371	-0.629	0.371	1.000	0.552	0.645	-	0.629
80	0.840	1.000	0.000	0.371	-0.629	0.371	1.000	0.552	0.645	-	0.629
81	0.850	1.000	0.000	0.371	-0.629	0.371	1.000	0.552	0.645	-	0.629
82	0.860	1.000	0.000	0.371	-0.629	0.371	1.000	0.552	0.645	-	0.629
83	0.870	1.000	0.000	0.355	-0.645	0.355	1.000	0.545	0.636	-	0.645
84	0.880	1.000	0.000	0.339	-0.661	0.339	1.000	0.539	0.627	-	0.661
85	0.890	1.000	0.000	0.323	-0.677	0.323	1.000	0.533	0.618	-	0.677
86	0.900	1.000	0.000	0.323	-0.677	0.323	1.000	0.533	0.618	-	0.677
87	0.910	1.000	0.000	0.323	-0.677	0.323	1.000	0.533	0.618	-	0.677
88	0.920	1.000	0.000	0.323	-0.677	0.323	1.000	0.533	0.618	-	0.677
89	0.930	1.000	0.000	0.323	-0.677	0.323	1.000	0.533	0.618	-	0.677
90	0.940	1.000	0.000	0.323	-0.677	0.323	1.000	0.533	0.618	-	0.677
91	0.950	1.000	0.000	0.323	-0.677	0.323	1.000	0.533	0.618	-	0.677
92	0.960	1.000	0.000	0.323	-0.677	0.323	1.000	0.533	0.618	-	0.677
93	0.970	1.000	0.000	0.323	-0.677	0.323	1.000	0.533	0.618	-	0.677
94	0.980	1.000	0.000	0.323	-0.677	0.323	1.000	0.533	0.618	-	0.677
95	0.990	1.000	0.000	0.323	-0.677	0.323	1.000	0.533	0.618	-	0.677
96	1.000	1.000	0.000	0.306	-0.694	0.306	1.000	0.527	0.609	-	0.694
97	1.010	1.000	0.000	0.306	-0.694	0.306	1.000	0.527	0.609	-	0.694
98	1.020	1.000	0.000	0.290	-0.710	0.290	1.000	0.522	0.600	-	0.710
99	1.030	1.000	0.000	0.290	-0.710	0.290	1.000	0.522	0.600	-	0.710
100	1.040	1.000	0.000	0.274	-0.726	0.274	1.000	0.516	0.591	-	0.726
101	1.050	1.000	0.000	0.274	-0.726	0.274	1.000	0.516	0.591	-	0.726
102	1.060	1.000	0.000	0.274	-0.726	0.274	1.000	0.505	0.583	-	0.726
103	1.070	1.000	0.000	0.258	-0.742	0.258	1.000	0.511	0.582	-	0.742
104	1.080	1.000	0.000	0.242	-0.758	0.242	1.000	0.505	0.573	-	0.758

Continued on the next page

Supplementary Table S4 *(continued)*

N. Cut-off	OD cut-off	Sp	1 - Sp	Se	Se-Sp	J	VPP	VPN	Accuracy	LR +	LR -
105	1.090	1.000	0.000	0.242	-0.758	0.242	1.000	0.505	0.573	-	0.758
106	1.100	1.000	0.000	0.226	-0.774	0.226	1.000	0.500	0.564	-	0.774
107	1.110	1.000	0.000	0.226	-0.774	0.226	1.000	0.500	0.564	-	0.774
108	1.120	1.000	0.000	0.194	-0.806	0.194	1.000	0.490	0.545	-	0.806
109	1.130	1.000	0.000	0.177	-0.823	0.177	1.000	0.485	0.536	-	0.823
110	1.140	1.000	0.000	0.177	-0.823	0.177	1.000	0.485	0.536	-	0.823
111	1.150	1.000	0.000	0.177	-0.823	0.177	1.000	0.485	0.536	-	0.823
112	1.160	1.000	0.000	0.177	-0.823	0.177	1.000	0.485	0.536	-	0.823
113	1.170	1.000	0.000	0.177	-0.823	0.177	1.000	0.485	0.536	-	0.823
114	1.180	1.000	0.000	0.177	-0.823	0.177	1.000	0.485	0.536	-	0.823
115	1.190	1.000	0.000	0.177	-0.823	0.177	1.000	0.485	0.536	-	0.823
116	1.200	1.000	0.000	0.161	-0.839	0.161	1.000	0.480	0.527	-	0.839
117	1.210	1.000	0.000	0.161	-0.839	0.161	1.000	0.480	0.527	-	0.839
118	1.220	1.000	0.000	0.161	-0.839	0.161	1.000	0.480	0.527	-	0.839
119	1.230	1.000	0.000	0.161	-0.839	0.161	1.000	0.480	0.527	-	0.839
120	1.240	1.000	0.000	0.145	-0.855	0.145	1.000	0.475	0.518	-	0.855
121	1.250	1.000	0.000	0.145	-0.855	0.145	1.000	0.475	0.518	-	0.855
122	1.260	1.000	0.000	0.145	-0.855	0.145	1.000	0.475	0.518	-	0.855
123	1.270	1.000	0.000	0.145	-0.855	0.145	1.000	0.475	0.518	-	0.855
124	1.280	1.000	0.000	0.145	-0.855	0.145	1.000	0.475	0.518	-	0.855
125	1.290	1.000	0.000	0.145	-0.855	0.145	1.000	0.475	0.518	-	0.855
126	1.300	1.000	0.000	0.145	-0.855	0.145	1.000	0.475	0.518	-	0.855
127	1.310	1.000	0.000	0.145	-0.855	0.145	1.000	0.475	0.518	-	0.855
128	1.320	1.000	0.000	0.145	-0.855	0.145	1.000	0.475	0.518	-	0.855
129	1.330	1.000	0.000	0.145	-0.855	0.145	1.000	0.475	0.518	-	0.855
130	1.340	1.000	0.000	0.145	-0.855	0.145	1.000	0.475	0.518	-	0.855
131	1.350	1.000	0.000	0.145	-0.855	0.145	1.000	0.475	0.518	-	0.855
132	1.360	1.000	0.000	0.145	-0.855	0.145	1.000	0.475	0.518	-	0.855
133	1.370	1.000	0.000	0.145	-0.855	0.145	1.000	0.475	0.518	-	0.855
134	1.380	1.000	0.000	0.129	-0.871	0.129	1.000	0.471	0.509	-	0.871
135	1.390	1.000	0.000	0.129	-0.871	0.129	1.000	0.471	0.509	-	0.871
136	1.400	1.000	0.000	0.129	-0.871	0.129	1.000	0.471	0.509	-	0.871
137	1.410	1.000	0.000	0.129	-0.871	0.129	1.000	0.471	0.509	-	0.871
138	1.420	1.000	0.000	0.129	-0.871	0.129	1.000	0.471	0.509	-	0.871
139	1.430	1.000	0.000	0.113	-0.887	0.113	1.000	0.466	0.500	-	0.887
140	1.440	1.000	0.000	0.081	-0.919	0.081	1.000	0.457	0.482	-	0.919
141	1.450	1.000	0.000	0.081	-0.919	0.081	1.000	0.457	0.482	-	0.919
142	1.460	1.000	0.000	0.065	-0.935	0.065	1.000	0.453	0.473	-	0.935
143	1.470	1.000	0.000	0.065	-0.935	0.065	1.000	0.453	0.473	-	0.935
144	1.480	1.000	0.000	0.065	-0.935	0.065	1.000	0.453	0.473	-	0.935
145	1.490	1.000	0.000	0.065	-0.935	0.065	1.000	0.453	0.473	-	0.935
146	1.500	1.000	0.000	0.048	-0.952	0.048	1.000	0.449	0.464	-	0.952
147	1.510	1.000	0.000	0.032	-0.968	0.032	1.000	0.444	0.455	-	0.968
148	1.520	1.000	0.000	0.032	-0.968	0.032	1.000	0.444	0.455	-	0.968
149	1.530	1.000	0.000	0.077	-0.923	0.077	1.000	0.444	0.469	-	0.923
150	1.540	1.000	0.000	0.032	-0.968	0.032	1.000	0.444	0.455	-	0.968
151	1.550	1.000	0.000	0.032	-0.968	0.032	1.000	0.444	0.455	-	0.968
152	1.560	1.000	0.000	0.032	-0.968	0.032	1.000	0.444	0.455	-	0.968
153	1.570	1.000	0.000	0.032	-0.968	0.032	1.000	0.444	0.455	-	0.968
154	1.580	1.000	0.000	0.032	-0.968	0.032	1.000	0.444	0.455	-	0.968
155	1.590	1.000	0.000	0.032	-0.968	0.032	1.000	0.444	0.455	-	0.968

Continued on the next page

Supplementary Table S4 *(continued)*

N. Cut-off	OD cut-off	Sp	1 - Sp	Se	Se-Sp	J	VPP	VPN	Accuracy	LR +	LR -
156	1.600	1.000	0.000	0.016	-0.984	0.016	1.000	0.440	0.445	-	0.984
157	1.610	1.000	0.000	0.016	-0.984	0.016	1.000	0.440	0.445	-	0.984
158	1.620	1.000	0.000	0.016	-0.984	0.016	1.000	0.440	0.445	-	0.984
159	1.630	1.000	0.000	0.016	-0.984	0.016	1.000	0.440	0.445	-	0.984
160	1.640	1.000	0.000	0.016	-0.984	0.016	1.000	0.440	0.445	-	0.984
161	1.650	1.000	0.000	0.016	-0.984	0.016	1.000	0.440	0.445	-	0.984
162	1.660	1.000	0.000	0.016	-0.984	0.016	1.000	0.440	0.445	-	0.984
163	1.670	1.000	0.000	0.016	-0.984	0.016	1.000	0.440	0.445	-	0.984
164	1.680	1.000	0.000	0.016	-0.984	0.016	1.000	0.440	0.445	-	0.984
165	1.690	1.000	0.000	0.016	-0.984	0.016	1.000	0.440	0.445	-	0.984
166	1.700	1.000	0.000	0.016	-0.984	0.016	1.000	0.440	0.445	-	0.984
167	1.710	1.000	0.000	0.016	-0.984	0.016	1.000	0.440	0.445	-	0.984
168	1.720	1.000	0.000	0.016	-0.984	0.016	1.000	0.440	0.445	-	0.984
169	1.730	1.000	0.000	0.016	-0.984	0.016	1.000	0.440	0.445	-	0.984
170	1.740	1.000	0.000	0.000	-1.000	0.000	-	0.436	0.436	-	1.000
171	1.750	1.000	0.000	0.000	-1.000	0.000	-	0.436	0.436	-	1.000
172	1.760	1.000	0.000	0.000	-1.000	0.000	-	0.436	0.436	-	1.000
173	1.770	1.000	0.000	0.000	-1.000	0.000	-	0.398	0.398	-	1.000
174	1.780	1.000	0.000	0.000	-1.000	0.000	-	0.436	0.436	-	1.000
175	1.790	1.000	0.000	0.000	-1.000	0.000	-	0.436	0.436	-	1.000
176	1.800	1.000	0.000	0.000	-1.000	0.000	-	0.436	0.436	-	1.000
177	1.810	1.000	0.000	0.000	-1.000	0.000	-	0.436	0.436	-	1.000

Supplementary Table S5. Reproducibility study. ELISA tests were performed in duplicate, twice a day, for 6 or 14 consecutive days (24 or 56 tests for each sample). PC: positive control; NC: negative control; samples: 1, 2, 3 (negative), 4 (positive). OD: optical density. n.d.: not determined.

**Samples**	**Day 1**				**Day 2**				**Day 3**				**Day 4**				**Day 5**			
	**OD 1**	**OD 2**	**OD 3**	**OD 4**	**OD 1**	**OD 2**	**OD 3**	**OD 4**	**OD 1**	**OD 2**	**OD 3**	**OD 4**	**OD 1**	**OD 2**	**OD 3**	**OD 4**	**OD 1**	**OD 2**	**OD 3**	**OD 4**
PC	2.689	2.666	2.671	2.677	2.639	2.657	2.661	2.654	2.626	2.635	2.677	2.654	2.675	2.673	2.643	2.639	2.645	2.658	2.665	2.671
NC	0.045	0.042	0.041	0.041	0.043	0.042	0.044	0.043	0.043	0.043	0.045	0.043	0.043	0.042	0.041	0.041	0.043	0.043	0.044	0.049
Sample 1	0.058	0.058	0.056	0.057	0.054	0.056	0.054	0.055	0.053	0.055	0.060	0.060	0.059	0.055	0.052	0.052	0.055	0.053	0.059	0.058
Sample 2	0.052	0.053	0.051	0.050	0.054	0.051	0.053	0.051	0.053	0.050	0.053	0.053	0.055	0.052	0.049	0.052	0.057	0.051	0.057	0.054
Sample 3	0.073	0.072	0.078	0.073	0.078	0.074	0.073	0.072	0.072	0.072	0.073	0.072	0.088	0.075	0.075	0.073	0.077	0.082	0.094	0.084
Sample 4	0.759	0.796	0.799	0.697	0.628	0.768	0.694	0.668	0.653	0.671	0.797	0.807	0.687	0.634	0.567	0.747	0.667	0.649	0.851	0.785

**Samples**	**Day 6**				**Day 7**				**Day 8**				**Day 9**				**Day 10**			
	**OD 1**	**OD 2**	**OD 3**	**OD 4**	**OD 1**	**OD 2**	**OD 3**	**OD 4**	**OD 1**	**OD 2**	**OD 3**	**OD 4**	**OD 1**	**OD 2**	**OD 3**	**OD 4**	**OD 1**	**OD 2**	**OD 3**	**OD 4**
PC	2.674	2.668	2.676	2.666	2.639	2.641	2.648	2.654	2.642	2.654	2.677	2.674	2.679	2.675	2.652	2.654	2.649	2.661	2.667	2.662
NC	0.044	0.043	0.042	0.045	n.d	n.d	n.d	n.d	n.d	n.d	n.d	n.d	n.d	n.d	n.d	n.d	n.d	n.d	n.d	n.d
Sample 1	0.061	0.061	0.060	0.056	n.d	n.d	n.d	n.d	n.d	n.d	n.d	n.d	n.d	n.d	n.d	n.d	n.d	n.d	n.d	n.d
Sample 2	0.054	0.054	0.052	0.052	n.d	n.d	n.d	n.d	n.d	n.d	n.d	n.d	n.d	n.d	n.d	n.d	n.d	n.d	n.d	n.d
Sample 3	0.073	0.074	0.079	0.075	n.d	n.d	n.d	n.d	n.d	n.d	n.d	n.d	n.d	n.d	n.d	n.d	n.d	n.d	n.d	n.d
Sample 4	0.676	0.675	0.711	0.663	n.d	n.d	n.d	n.d	n.d	n.d	n.d	n.d	n.d	n.d	n.d	n.d	n.d	n.d	n.d	n.d

**Samples**	**Day 11**				**Day 12**				**Day 13**				**Day 14**			
	**OD 1**	**OD 2**	**OD 3**	**OD 4**	**OD 1**	**OD 2**	**OD 3**	**OD 4**	**OD 1**	**OD 2**	**OD 3**	**OD 4**	**OD 1**	**OD 2**	**OD 3**	**OD 4**
PC	2.664	2.660	2.676	2.671	2.678	2.673	2.652	2.657	2.675	2.670	2.665	2.669	2.672	2.679	2.669	2.672
NC	n.d	n.d	n.d	n.d	n.d	n.d	n.d	n.d	n.d	n.d	n.d	n.d	n.d	n.d	n.d	n.d
Sample 1	n.d	n.d	n.d	n.d	n.d	n.d	n.d	n.d	n.d	n.d	n.d	n.d	n.d	n.d	n.d	n.d
Sample 2	n.d	n.d	n.d	n.d	n.d	n.d	n.d	n.d	n.d	n.d	n.d	n.d	n.d	n.d	n.d	n.d
Sample 3	n.d	n.d	n.d	n.d	n.d	n.d	n.d	n.d	n.d	n.d	n.d	n.d	n.d	n.d	n.d	n.d
Sample 4	n.d	n.d	n.d	n.d	n.d	n.d	n.d	n.d	n.d	n.d	n.d	n.d	n.d	n.d	n.d	n.d

Supplementary Table S6. Intra- and inter-assay coefficient of variation of FCoVCHECK Ab ELISA. PC: positive control; NC: negative control; samples: 1, 2, 3 (negative), 4 (positive). %CV: coefficient of variation (%). MIN: minimum %CV; MAX: maximum %CV; n: number of assays; n.d.: not determined.

Day of analysis	N. assays	Intra-assay %CV						Inter-assay %CV					
		PC	NC	Sample 1	Sample 2	Sample 3	Sample 4	PC	NC	Sample 1	Sample 2	Sample 3	Sample 4
Day 1	1	0.607	4.877	0.000	1.347	0.975	3.365	0.383	2.438	0.626	1.374	2.829	6.504
	2	0.159	0.000	1.252	1.400	4.683	9.642						
Day 2	3	0.481	1.664	2.571	4.041	3.722	14.183	0.333	1.645	1.934	3.380	2.348	8.441
	4	0.186	1.626	1.297	2.720	0.975	2.700						
Day 3	5	0.242	0.000	2.619	4.119	0.000	1.923	0.426	1.607	1.309	2.060	0.488	1.402
	6	0.610	3.214	0.000	0.000	0.975	0.882						
Day 4	7	0.053	1.664	4.962	3.965	11.279	5.674	0.080	0.832	2.481	4.083	6.595	12.523
	8	0.107	0.000	0.000	4.201	1.911	19.373						
Day 5	9	0.347	0.000	2.619	7.857	4.447	1.934	0.253	3.802	1.914	5.839	6.196	3.820
	10	0.159	7.603	1.209	3.822	7.945	5.705						
Day 6	11	0.159	1.626	0.000	0.000	0.962	0.105	0.212	3.251	2.438	0.000	2.318	2.523
	12	0.265	4.877	4.877	0.000	3.673	4.940						
Day 7	13	0.054	n.d.	n.d.	n.d.	n.d.	n.d.	0.107	n.d	n.d	n.d	n.d	n.d
	14	0.160	n.d.	n.d.	n.d.	n.d.	n.d.						
Day 8	15	0.320	n.d.	n.d.	n.d.	n.d.	n.d.	0.200	n.d	n.d	n.d	n.d	n.d
	16	0.079	n.d.	n.d.	n.d.	n.d.	n.d.						
Day 9	17	0.106	n.d.	n.d.	n.d.	n.d.	n.d.	0.079	n.d	n.d	n.d	n.d	n.d
	18	0.053	n.d.	n.d.	n.d.	n.d.	n.d.						
Day 10	19	0.320	n.d.	n.d.	n.d.	n.d.	n.d.	0.226	n.d	n.d	n.d	n.d	n.d
	20	0.133	n.d.	n.d.	n.d.	n.d.	n.d.						
Day 11	21	0.106	n.d.	n.d.	n.d.	n.d.	n.d.	0.119	n.d	n.d	n.d	n.d	n.d
	22	0.132	n.d.	n.d.	n.d.	n.d.	n.d.						
Day 12	23	0.132	n.d.	n.d.	n.d.	n.d.	n.d.	0.133	n.d	n.d	n.d	n.d	n.d
	24	0.133	n.d.	n.d.	n.d.	n.d.	n.d.						
Day 13	25	0.132	n.d.	n.d.	n.d.	n.d.	n.d.	0.119	n.d	n.d	n.d	n.d	n.d
	26	0.106	n.d.	n.d.	n.d.	n.d.	n.d.						
Day 14	27	0.185	n.d.	n.d.	n.d.	n.d.	n.d.	0.132	n.d	n.d	n.d	n.d	n.d
	28	0.079	n.d.	n.d.	n.d.	n.d.	n.d.						
**MIN**		**0.053**	**0.000**	**0.000**	**0.000**	**0.000**	**0.105**	**0.079**	**0.832**	**0.626**	**0.000**	**0.488**	**1.402**
**MAX**		**0.610**	**7.603**	**4.962**	**7.857**	**11.279**	**19.373**	**0.426**	**3.802**	**2.481**	**5.839**	**6.595**	**12.523**

Supplementary Table S7. Comparison results of FCoVCHECK Ab ELISA, ImmunoComb and INgezim Corona Felino ELISA. Number of samples analysed: 110. All discordant results are highlighted in bold, while those ones only with ImmunoComb are marked with an asterisk. CombScale scores: S0 and S1 (negative); S2 (weak positive or borderline, FIP improbable); S3 and S4 (positive, FIP possible); ≥ S5 (strong positive, high probability of FIP). OD: optical density. POS: positive; NEG: negative. GSD: Gold Standard Diagnostics.

N. sample	IFAT	FCoVCHECK Ab ELISA (Agrolabo)		INgezim Corona Felino (GSD)		ImmunoComb (Biogal)
		OD	Results	OD	Results	CombScale
**1**	**NEG**	0.105	NEG	**0.439**	**POS**	S2
**2**	**NEG**	0.157	NEG	**0.580**	**POS**	S2
3	POS	0.694	POS	0.760	POS	S5
4	POS	1.434	POS	1.911	POS	S6
**5**	**NEG**	0.262	NEG	**0.352**	**POS**	S1
**6**	**NEG**	0.117	NEG	**0.502**	**POS**	S2
**7**	**NEG**	0.141	NEG	**0.463**	**POS**	S2
8	POS	0.865	POS	1.135	POS	S3
9	POS	0.371	POS	0.450	POS	S3
**10**	**NEG**	0.098	NEG	**0.320**	**POS**	S2
11	POS	0.551	POS	0.485	POS	S3
12	NEG	0.086	NEG	0.297	NEG	S2
13	POS	0.308	POS	0.475	POS	S3
14	POS	0.735	POS	0.640	POS	S3
**15***	**NEG**	0.068	NEG	0.234	NEG	**S4**
16	POS	0.494	POS	0.443	POS	S3
17	POS	0.591	POS	0.712	POS	S4
18	POS	0.385	POS	0.437	POS	S3
19	POS	0.662	POS	0.864	POS	S5
20	POS	0.447	POS	0.489	POS	S4
**21**	**NEG**	0.189	NEG	**0.256**	**POS**	S1
22	POS	0.567	POS	0.720	POS	S3
**23***	**NEG**	0.289	DOUBTFUL	**0.519**	**POS**	**S3**
24	POS	1.455	POS	1.625	POS	S5
**25**	**NEG**	0.088	NEG	**0.199**	**POS**	S2
26	POS	0.316	POS	0.746	POS	S5
27	POS	1.599	POS	1.651	POS	S6
**28**	**NEG**	0.253	NEG	**0.201**	**POS**	S2
29	POS	1.116	POS	0.694	POS	S5
**30**	**NEG**	0.120	NEG	**0.287**	**POS**	S0
31	POS	0.660	POS	1.093	POS	S5
32	POS	0.309	POS	1.145	POS	S5
**33***	**NEG**	0.145	NEG	**0.480**	**POS**	**S5**
**34**	**NEG**	0.215	NEG	**0.234**	**POS**	S2
35	POS	0.768	POS	0.488	POS	S4
36	POS	0.416	POS	0.997	POS	S5
**37**	**POS**	0.085	NEG	**0.232**	**POS**	S2
38	POS	1.507	POS	1.822	POS	S6
**39***	**NEG**	0.066	NEG	**0.442**	**POS**	**S4**
40	POS	0.876	POS	0.839	POS	S3
41	POS	0.311	POS	0.354	POS	S3
42	POS	0.349	POS	0.892	POS	S5
43	POS	0.363	POS	0.387	POS	S3
**44**	**NEG**	0.205	NEG	**0.428**	**POS**	S2
45	POS	0.318	POS	0.538	POS	S4
**46***	**POS**	0.088	NEG	**0.885**	**POS**	**S4**
47	POS	1.421	POS	0.984	POS	S6
48	POS	0.305	POS	0.323	POS	S3
49	POS	1.124	POS	0.826	POS	S5
50	POS	1.095	POS	0.944	POS	S5

Continued on the next page

Supplementary Table S7 *(continued)*

N. sample	IFAT	FCoVCHECK Ab ELISA (Agrolabo)		INgezim Corona Felino (GSD)		ImmunoComb (Biogal)
		OD	Results	OD	Results	CombScale
51	POS	1.011	POS	0.823	POS	S5
52	POS	0.388	POS	0.344	POS	S3
53	POS	1.195	POS	1.359	POS	S5
**54***	**NEG**	0.108	NEG	**0.453**	**POS**	**S5**
55	POS	0.435	POS	0.406	POS	S3
56	POS	0.549	POS	0.658	POS	S4
57	POS	0.625	POS	0.908	POS	S5
58	POS	0.519	POS	0.933	POS	S3
59	POS	0.650	POS	0.467	POS	S3
60	POS	0.313	POS	0.461	POS	S3
61	POS	1.376	POS	1.526	POS	S5
62	POS	0.883	POS	0.739	POS	S5
**63***	**NEG**	0.083	NEG	**0.426**	**POS**	**S4**
64	NEG	0.089	NEG	0.274	NEG	S1
65	POS	0.337	POS	0.442	POS	S4
66	POS	0.335	POS	0.596	POS	S3
67	POS	0.808	POS	1.171	POS	S5
68	POS	0.323	POS	0.446	POS	S3
69	POS	0.995	POS	1.245	POS	S5
70	POS	1.032	POS	1.281	POS	S5
71	POS	0.727	POS	1.391	POS	S5
72	POS	1.497	POS	1.628	POS	S5
73	POS	1.064	POS	1.538	POS	S5
74	POS	0.575	POS	0.754	POS	S3
75	NEG	0.105	NEG	0.221	NEG	S2
76	NEG	0.129	NEG	0.169	NEG	S2
77	NEG	0.079	NEG	0.065	NEG	S0
78	NEG	0.097	NEG	0.105	NEG	S1
79	NEG	0.082	NEG	0.147	NEG	S2
80	NEG	0.070	NEG	0.079	NEG	S0
81	NEG	0.061	NEG	0.062	NEG	S0
82	NEG	0.052	NEG	0.050	NEG	S0
83	NEG	0.237	NEG	0.226	NEG	S1
84	NEG	0.053	NEG	0.055	NEG	S0
85	NEG	0.096	NEG	0.098	NEG	S0
86	NEG	0.066	NEG	0.088	NEG	S0
87	NEG	0.063	NEG	0.088	NEG	S0
88	NEG	0.062	NEG	0.160	NEG	S2
89	NEG	0.067	NEG	0.103	NEG	S0
90	NEG	0.123	NEG	0.155	NEG	S1
91	NEG	0.051	NEG	0.081	NEG	S0
92	NEG	0.081	NEG	0.156	NEG	S2
93	NEG	0.063	NEG	0.120	NEG	S0
94	NEG	0.083	NEG	0.182	NEG	S2
95	NEG	0.090	NEG	0.121	NEG	S2
96	NEG	0.180	NEG	0.170	NEG	S2
97	NEG	0.067	NEG	0.093	NEG	S0
98	POS	0.110	NEG	0.147	NEG	S1
99	NEG	0.062	NEG	0.125	NEG	S1
100	NEG	0.241	NEG	0.201	NEG	S1
101	NEG	0.070	NEG	0.112	NEG	S0
102	NEG	0.075	NEG	0.101	NEG	S0
103	POS	0.070	NEG	0.202	NEG	S0

Continued on the next page

Supplementary Table S7 *(continued)*

N. sample	IFAT	FCoVCHECK Ab ELISA (Agrolabo)		INgezim Corona Felino (GSD)		ImmunoComb (Biogal)
		OD	Results	OD	Results	CombScale
104	NEG	0.072	NEG	0.100	NEG	S0
105	POS	1.236	POS	1.845	POS	S5
106	POS	1.112	POS	1.472	POS	S5
107	POS	1.074	POS	1.433	POS	S5
108	POS	0.304	POS	0.234	POS	S3
109	POS	1.431	POS	1.081	POS	S6
110	POS	1.733	POS	1.814	POS	S6

Supplementary Table S8. Titration assay performed by FCoVCHECK Ab ELISA and INgezim Corona Felino ELISA. Number of positive samples tested: 21. Samples were tested at dilution from 1:200 to 1:12,800. Fields in bold indicate the OD value corresponding to the antibody titer. OD cut-off values of INgezim Corona Felino ELISA and FCoVCHECK Ab ELISA were 0.308 and 0.255, respectively. n.d: not determined.

Samples	INgezim Corona Felino ELISA (Gold Standard Diagnostics)				FCoVCHECK Ab ELISA (Agrolabo)			
	1:200	1:3,200	1:6,400	1:12,800	1:200	1:3,200	1:6,400	1:12,800
Sample 1	**0.760**	0.119	0.077	0.059	**0.592**	0.113	0.077	0.068
Sample 2	1.911	**0.371**	0.218	0.121	1.324	**0.310**	0.202	0.145
Sample 3	**0.640**	0.125	0.085	0.064	**0.721**	0.146	0.094	0.081
Sample 4	**0.443**	0.075	n.d.	n.d.	**0.413**	0.073	0.062	0.056
Sample 5	**0.712**	0.097	n.d.	n.d.	**0.362**	0.070	0.058	0.058
Sample 6	**0.864**	0.167	n.d.	n.d.	**0.562**	0.144	0.101	0.078
Sample 7	**0.720**	0.132	n.d.	n.d.	**0.465**	0.094	0.071	0.065
Sample 8	1.885	0.787	0.499	**0.318**	1.452	0.446	**0.287**	0.209
Sample 9	1.863	0.795	**0.469**	0.300	1.712	0.565	**0.357**	0.214
Sample 10	1.988	0.762	**0.459**	0.304	1.769	0.518	**0.365**	0.230
Sample 11	**1.341**	0.277	0.168	0.101	**1.176**	0.254	0.165	0.114
Sample 12	**0.406**	0.060	n.d.	n.d.	**0.269**	0.066	0.052	0.050
Sample 13	1.599	**0.358**	0.210	0.134	1.500	0.429	**0.274**	0.167
Sample 14	**1.245**	0.199	n.d.	n.d.	**0.811**	0.136	0.098	0.076
Sample 15	1.355	0.501	**0.321**	0.218	1.216	0.442	**0.273**	0.199
Sample 16	1.403	**0.411**	0.248	0.166	**0.723**	0.227	0.154	0.111
Sample 17	1.863	0.405	**0.359**	0.151	1.781	0.535	**0.332**	0.248
Sample 18	1.586	**0.416**	0.252	0.153	1.123	**0.311**	0.188	0.133
Sample 19	2.159	0.648	**0.401**	0.236	1.391	**0.344**	0.222	0.138
Sample 20	1.769	0.551	**0.365**	0.213	1.183	0.415	**0.283**	0.194
Sample 21	1.458	**0.400**	0.265	0.173	1.298	**0.309**	0.197	0.136

Supplementary Table S9. Accelerated stability study. At each time of analysis (T_0 – T_6) samples were tested in duplicate, then the mean OD values and percentage remaining activities (%RA) were calculated. PC: positive control; NC: negative control; samples: 1, 2,3 (negative), 4 (positive).

**Samples**	**T_0**				**T_1**				**T_2**				**T_3**			
	**OD 1**	**OD 2**	**Mean**	**%RA**	**OD 1**	**OD 2**	**Mean**	**%RA**	**OD 1**	**OD 2**	**Mean**	**%RA**	**OD 1**	**OD 2**	**Mean**	**%RA**
PC	2.877	2.856	2.867	100.00	2.713	2.777	2.745	95.76	2.626	2.624	2.625	91.58	2.622	2.639	2.631	91.77
NC	0.042	0.044	0.043	100.00	0.048	0.045	0.047	108.14	0.047	0.049	0.048	111.63	0.049	0.046	0.048	110.47
Sample 1	0.048	0.048	0.048	100.00	0.049	0.049	0.049	102.08	0.050	0.052	0.051	106.25	0.051	0.048	0.050	103.13
Sample 2	0.056	0.057	0.057	100.00	0.058	0.059	0.059	103.54	0.061	0.065	0.063	111.50	0.059	0.059	0.059	104.42
Sample 3	0.173	0.175	0.174	100.00	0.177	0.166	0.172	98.56	0.173	0.172	0.173	99.14	0.151	0.148	0.150	85.92
Sample 4	0.526	0.513	0.520	100.00	0.533	0.544	0.539	103.66	0.591	0.509	0.550	105.87	0.428	0.492	0.460	88.55

**Samples**	**T_4**				**T_5**				**T_6**			
	**OD 1**	**OD 2**	**Mean**	**%RA**	**OD 1**	**OD 2**	**Mean**	**%RA**	**OD 1**	**OD 2**	**Mean**	**%RA**
PC	2.667	2.657	2.662	92.87	2.601	2.611	2.606	90.91	2.547	2.556	2.552	89.01
NC	0.047	0.048	0.048	110.47	0.046	0.048	0.047	109.30	0.045	0.044	0.045	103.49
Sample 1	0.051	0.055	0.053	110.42	0.047	0.049	0.048	100.00	0.047	0.050	0.049	101.04
Sample 2	0.060	0.061	0.061	107.08	0.055	0.060	0.058	101.77	0.055	0.056	0.056	98.23
Sample 3	0.161	0.153	0.157	90.23	0.127	0.146	0.137	78.45	0.150	0.146	0.148	85.06
Sample 4	0.453	0.470	0.462	88.84	0.424	0.439	0.432	83.06	0.447	0.440	0.444	85.37

Supplementary Table S10. Real-time stability study. At each time of analysis (T_0 – T_{12}) samples were tested in duplicate, then the mean OD values and percentage remaining activities (%RA) were calculated. PC: positive control; NC: negative control. Samples: 1, 2 (negative); 3, 4, 5 (positive).

Samples	T_0				T_1				T_2				T_3				T_4			
	**OD 1**	**OD 2**	**Mean**	**%RA**	**OD 1**	**OD 2**	**Mean**	**%RA**	**OD 1**	**OD 2**	**Mean**	**%RA**	**OD 1**	**OD 2**	**Mean**	**%RA**	**OD 1**	**OD 2**	**Mean**	**%RA**
PC	2.877	2.856	2.867	100.00	2.913	2.929	2.921	101.90	2.859	2.858	2.859	99.72	2.854	2.855	2.855	99.58	2.867	2.857	2.862	99.84
NC	0.049	0.047	0.048	100.00	0.047	0.046	0.047	96.88	0.050	0.049	0.050	103.13	0.041	0.049	0.045	93.75	0.048	0.048	0.048	100.00
Sample 1	0.051	0.059	0.055	100.00	0.057	0.053	0.055	100.00	0.051	0.056	0.054	97.27	0.053	0.049	0.051	92.73	0.059	0.053	0.056	101.82
Sample 2	0.062	0.065	0.064	100.00	0.064	0.070	0.067	105.51	0.062	0.069	0.066	103.15	0.055	0.060	0.058	90.55	0.063	0.065	0.064	100.79
Sample 3	0.535	0.551	0.543	100.00	0.509	0.514	0.512	94.20	0.506	0.507	0.507	93.28	0.548	0.563	0.556	102.30	0.566	0.569	0.568	104.51
Sample 4	0.830	0.828	0.829	100.00	0.802	0.862	0.832	100.36	0.794	0.791	0.793	95.60	0.769	0.795	0.782	94.33	0.814	0.807	0.811	97.77
Sample 5	1.222	1.225	1.224	100.00	1.193	1.197	1.195	97.67	1.183	1.187	1.185	96.85	1.154	1.158	1.156	94.48	1.164	1.146	1.155	94.40

Samples	T_5				T_6				T_7 (3 months)				T_8 (6 months)				T_9 (9 months)			
	**OD 1**	**OD 2**	**Mean**	**%RA**	**OD 1**	**OD 2**	**Mean**	**%RA**	**OD 1**	**OD 2**	**Mean**	**%RA**	**OD 1**	**OD 2**	**Mean**	**%RA**	**OD 1**	**OD 2**	**Mean**	**%RA**
PC	2.905	2.901	2.903	101.27	2.880	2.884	2.882	100.54	2.853	2.869	2.861	99.81	2.789	2.791	2.790	97.33	2.711	2.783	2.747	95.83
NC	0.050	0.051	0.051	105.21	0.049	0.048	0.049	101.04	0.048	0.046	0.047	97.92	0.045	0.047	0.046	95.83	0.050	0.050	0.050	104.17
Sample 1	0.061	0.054	0.058	104.55	0.050	0.053	0.052	93.64	0.057	0.054	0.056	100.91	0.053	0.057	0.055	100.00	0.051	0.054	0.053	95.46
Sample 2	0.068	0.059	0.064	100.00	0.061	0.058	0.060	93.70	0.065	0.063	0.064	100.79	0.062	0.064	0.063	99.21	0.058	0.069	0.064	100.00
Sample 3	0.564	0.561	0.563	103.59	0.543	0.539	0.541	99.63	0.540	0.538	0.539	99.26	0.539	0.531	0.535	98.53	0.486	0.481	0.484	89.04
Sample 4	0.793	0.799	0.796	96.02	0.772	0.726	0.749	90.35	0.828	0.799	0.814	98.13	0.799	0.810	0.805	97.05	0.708	0.682	0.695	83.84
Sample 5	1.169	1.177	1.173	95.87	1.174	1.167	1.171	95.67	1.224	1.199	1.212	99.02	1.210	1.211	1.211	98.94	1.022	1.051	1.037	84.72

Samples	T_{10} (12 months)				T_{11} (15 months)				T_{12} (18 months)			
	**OD 1**	**OD 2**	**Mean**	**%RA**	**OD 1**	**OD 2**	**Mean**	**%RA**	**OD 1**	**OD 2**	**Mean**	**%RA**
PC	2.517	2.544	2.531	88.28	2.501	2.511	2.506	87.42	2.389	2.391	2.390	83.38
NC	0.048	0.047	0.048	98.96	0.045	0.046	0.046	94.79	0.044	0.044	0.044	91.67
Sample 1	0.055	0.050	0.053	95.46	0.049	0.047	0.048	87.27	0.045	0.046	0.046	82.73
Sample 2	0.058	0.056	0.057	89.76	0.051	0.053	0.052	81.89	0.052	0.051	0.052	81.10
Sample 3	0.403	0.414	0.409	75.23	0.400	0.402	0.401	73.85	0.389	0.391	0.390	71.82
Sample 4	0.645	0.654	0.650	78.35	0.621	0.624	0.623	75.09	0.602	0.605	0.604	72.80
Sample 5	0.971	0.978	0.975	79.65	0.968	0.965	0.967	79.00	0.884	0.887	0.886	72.37
